# Retraction: IL-26 Promotes the Proliferation and Survival of Human Gastric Cancer Cells by Regulating the Balance of STAT1 and STAT3 Activation

**DOI:** 10.1371/journal.pone.0274980

**Published:** 2022-09-15

**Authors:** The PLOS ONE Editors

Following the publication of this article [1], concerns were raised regarding the results presented in Figs 1, 2, 3, 4, and 5. Specifically,

- In Fig 1C, the IL-20R1 N.C. panel appears to partially overlap with the STAT3 (S727) N.C. panel.
- The FACS data presented in the panels listed below appear more similar than would be expected from independent samples:
  ○ Fig 2A: a1, a2, a3, a4, a5, a6, a7, b1, b2.
  ○ Fig 3B: MKN45, MKN45+1ng/ml IL-26, MKN45+10ng/ml, MKN45+50ng/ml IL-26, SGC7901, SGC7901+1ng/ml IL-26, SGC7901+10ng/ml IL-26, and SGC7901+50ng/ml IL-26.
  ○ Fig 5C: MKN**cs**+10ng/ml IL-26, MKN-STAT1siRNA+10ng/nl IL-26, SGC7901**cs**+10ng/ml IL-26, SGC7901-STAT1siRNA+10ng/ml IL-26, and SGC7901-STAT3siRNA+10ng/ml IL-26.
- Irregularities have been detected in the background of the following panels
  ○ Fig 4A IL-20R1 panel
  ○ Fig 4B Bcl-xl panel
  ○ Fig 5A STAT1 panel

The authors did not respond to the editorial queries about these concerns, nor did they provide the original data underlying the results presented in this article. In the absence of the original data underlying the published results, these issues cannot be resolved.

In light of the concerns affecting multiple figure panels that question the integrity of these data, the *PLOS ONE* Editors retract this article.

All authors either did not respond directly or could not be reached.
